# Severe Symptomatic Hypermagnesemia Associated with Over-the-Counter Laxatives in a Patient with Renal Failure and Sigmoid Volvulus

**DOI:** 10.1155/2014/560746

**Published:** 2014-01-06

**Authors:** Talal Khairi, Syed Amer, Samuel Spitalewitz, Lutfi Alasadi

**Affiliations:** ^1^Department of Nephrology, Brookdale University Hospital and Medical Center, Brooklyn, NY 11212, USA; ^2^Department of Internal Medicine, Brookdale University Hospital and Medical Center, Brooklyn, NY 11212, USA

## Abstract

Hypermagnesemia is an uncommon but a potentially serious clinical condition. Over-the-counter magnesium containing products are widely used as antacids or laxatives. Although generally well tolerated in patients with normal renal function, their unsupervised use in the elderly can result in severe symptomatic hypermagnesemia, especially in those patients with concomitant renal failure and bowel disorders. We report a case of severe symptomatic hypermagnesemia associated with over-the-counter laxatives in a 70-year-old male patient with renal failure and sigmoid volvulus, who was successfully treated with hemodialysis.

## 1. Case History and Hospital Course

A 70-year-old male, with past medical history of chronic obstructive pulmonary disease (COPD), long standing hypertension, and congestive heart failure presented with chest tightness and dyspnea, which was progressively getting worse over the past two days. On physical examination, he had labored breathing for which he was subsequently intubated. He also had diffusely tender and distended abdomen. He had trace edema in his lower extremities and absent deep tendon reflexes. Labs were significant for a white cell count of 21300, blood urea nitrogen (BUN) of 44 mg/dL with a creatinine of 2.5 mg/dL, B-type natriuretic peptide (BNP) of 1440, and magnesium of 11 mg/dL. EKG showed a pace maker rhythm. Chest radiograph revealed pulmonary congestion. Abdominal radiograph demonstrated distended loops of bowel with prominent air fluid levels in the left lower quadrant suggestive of sigmoid volvulus.

Nephrology service was consulted for acute renal failure and hypermagnesemia. On further questioning, the patient' family revealed that he was taking about 4 doses of milk of magnesia daily, over the past 5 days for constipation. He was then started on furosemide drip to manage his acute renal failure which seemed most likely due to his poorly controlled congestive heart failure.

Subsequently, a flexible sigmoidoscopy was performed which revealed sigmoid volvulus with ulcerated friable area consistent with ischemic injury. Surgical intervention was deferred for management of hypermagnesemia. He was hemodialysed in two sessions of 3 hours each, via a temporary dialysis catheter. Subsequently, his magnesium level and renal function normalized. He was then taken for surgery and intraoperative findings confirmed sigmoid volvulus with ischemic bowel, for which he underwent sigmoid resection and placement of cecostomy. His postoperative course was uneventful.

## 2. Discussion

Magnesium is the second most common intracellular divalent cation which functions as an allosteric modulator of several enzymes and is also responsible for bridging structurally distinct molecules [1]. One of the important functions of magnesium is to balance calcium levels in the intracellular fluids which in turn effect the neuromuscular junctions and the cardiovascular and central nervous systems [2]. Normal total plasma magnesium concentration varies in a narrow range (1.7–2.4 mg/dL). The kidney plays a crucial role in maintaining magnesium homeostasis. In the kidney about 10 percent of filtered magnesium is absorbed in the proximal tubule and 50 to 70 percent of the filtered magnesium is passively reabsorbed in the cortical aspect of the thick ascending limb of Henle [3].

There are 3 main processes via which hypermagnesemia can occur. The mechanism of hypermagnesemia includes-decreased elimination (as in case of renal insufficiency), magnesium overdose (as an infusion in treatment of severe preeclampsia/eclampsia or oral administration for use as an antacid or laxative), increased absorption as in case of impaired motility (drug induced opiates, anticholinergics; mechanical bowel obstruction). In our patient all the three mechanisms implicated for hypermagnesemia were operational: he had decreased elimination as a consequence of acute kidney injury and ingestion of multiple oral doses of milk of magnesia and had increased magnesium absorption secondary to the mechanical bowel obstruction from the sigmoid volvulus.

Presentation of hypermagnesemia depends on serum magnesium concentration (Table 1).

Treatment approach for hypermagnesemia is dependent on the renal function, magnesium concentration, and clinical symptoms. Two important treatment principles in management of acute severe hypermagnesemia are inhibition of action of magnesium and elimination of magnesium from blood [4]. The former effect is achieved by administration of intravenous calcium that serves as an magnesium antagonist, to reverse the neuromuscular and cardiac effects of hypermagnesemia [5] and the latter effect is achieved by hemodialysis. Hemodialysis, with its higher flow rates, would be preferred in acute magnesium intoxication because it can lower serum magnesium levels more efficiently to safer levels when compared with peritoneal dialysis. Our patient responded well to hemodialysis and his magnesium levels normalized after two 3-hour hemodialysis sessions.

## 3. Conclusion

Severe symptomatic hypermagnesemia may result from unsupervised use of magnesium containing over-the-counter laxatives, which are often considered benign. In the setting of renal insufficiency, severe magnesium toxicity can develop especially in the elderly. Presentation can mimic various medical conditions such as sepsis or respiratory failure and hence the clinician should have a high degree of suspicion to order further testing to successfully diagnose this potentially life threatening electrolyte disturbance. Once diagnosed, treatment with rapid supportive measures—intravenous calcium, fluids, loop diuretics, and urgent hemodialys—is is highly effective in preventing significant morbidity and mortality. Clinicians should be cautious while prescribing magnesium based laxatives in the elderly population.

## Figures and Tables

**Table 1:** Dose related manifestations of hypermagnesemia [6].

Serum magnesium level (mg/dL)	Dose related effects
1.7–2.4	Normal serum levels
5–8	Nausea, headache, light headedness, cutaneous flushing
9–12	Absent deep tendon reflexes, somnolence, hypotension
12–15	Sinoatrial and atrioventricular block, muscle paralysis, hypoventilation
>15	Cardiac asystole, respiratory arrest, coma
